# Supplementary material for: Clinical Significance of Gamma-Glutamyltranspeptidase Combined with Carbohydrate-Deficient Transferrin for the Assessment of Excessive Alcohol Consumption in Patients with Alcoholic Cirrhosis
Source: Medicines (Basel). 2021 Jul 19;8(7):39. doi: 10.3390/medicines8070039 (PMC8307258; doi:10.3390/medicines8070039)
Supplement: Supplementary file 1 [file medicines-08-00039-s001.zip › medicines-1305725-supplementary.pdf]

# Supplementary Materials: Clinical Significance of Gamma-Glutamyltranspeptidase Combined with Carbohydrate-Deficient Transferrin for the Assessment of Excessive Alcohol Consumption in Patients with Alcoholic Cirrhosis

**Akihiko Shibamoto, Tadashi Namisaki, Junya Suzuki, Takahiro Kubo, Satoshi Iwai, Fumimasa Tomooka, Soichi Takeda, Yuki Fujimoto, Masahide Enomoto, Koji Murata, Takashi Inoue, Koji Ishida, Hiroyuki Ogawa, Hirotetsu Takagi, Daisuke Kaya, Yuki Tsuji, Takahiro Ozutsumi, Yukihisa Fujinaga, Masanori Furukawa, Norihisa Nishimura, Yasuhiko Sawada, Koh Kitagawa, Shinya Sato, Hiroaki Takaya, Kosuke Kaji, Naotaka Shimozato, Hideto Kawaratani, Kei Moriya, Takemi Akahane, Akira Mitoro and Hitoshi Yoshiji**

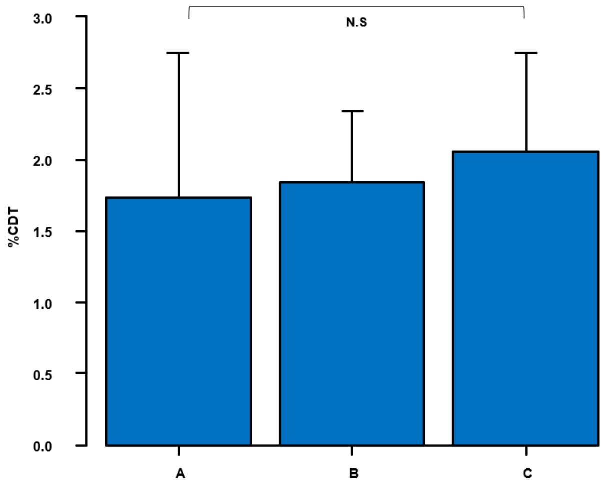


**Figure S1.** Relationship between Child-pugh classification and levels of %carbohydrate deficient transferrin.
